# Gum‐Assisted Magnesium Oxide Nanoparticles Using Guar Extract Focusing on Their Bioactivities

**DOI:** 10.1049/nbt2/9924353

**Published:** 2025-12-26

**Authors:** Asma Sepahdar, Matin Kordestani, Maryam Karkhane, Saeed Bahadori, Suresh Ghotekar, Pegah Shakib, Abdolrazagh Marzban

**Affiliations:** ^1^ Razi Herbal Medicines Research Center, Lorestan University of Medical Sciences, Khorramabad, Iran, lums.ac.ir; ^2^ Department of Restorative Dentistry, School of Dentistry, Lorestan University of Medical Sciences, Khorramabad, Iran, lums.ac.ir; ^3^ Student Research Committee, Department of Tissue Engineering and Applied Cell Sciences, School of Advanced Technologies in Medicine, Shahid Beheshti University of Medical Sciences, Tehran, Iran, sbmu.ac.ir; ^4^ Centre for Herbal Pharmacology and Environmental Sustainability, Chettinad Hospital and Research Institute, Chettinad Academy of Research and Education, Kelambakkam, Tamil Nadu, 603103, India

**Keywords:** antibiofilm, denal carries, *Enterococcus feacalis*, guar gum, MgO NPs

## Abstract

The study aims to fabricate eco‐friendly, biogenic magnesium oxide nanoparticles (MgO NPs) mediated by ethanol‐guar gum extract, which acts as both a reducing and coating/stabilizing agent. The prepared MgO NPs were first synthesized and characterized by various analytical techniques, including UV–visible, FTIR spectroscopy, SEM‐energy‐dispersive X‐ray spectroscopy (EDS) mapping, and X‐ray diffraction (XRD) crystallography. Bioactivity studies included antibacterial studies focusing on the inhibition of a dental caries‐causing pathogen, *Enterococcus faecalis*, by MIC, MBC, well diffusion (WD) agar, antibiofilm, and time‐kill (TK) assays. Furthermore, the antioxidant activity and cytotoxicity of MgO NPs were examined. A bacterial adherence study was conducted as the main aim by exposing the bacteria to human teeth in vitro. Findings demonstrated that biogenic MgO NPs were successfully synthesized with flaky morphologies, with an average size of 20–30 nm and the desired purity. FTIR showed possible functional groups, confirming the involvement of guar metabolites in NP formation. The XRD pattern elucidated the crystalline phase of MgO NPs to be a cubic (FCC) periclase structure with a crystallite size of 16.5 nm. Antibacterial experiments showed that MgO NPs had a moderate effect on *Enterococcus faecalis*, with MIC and MBC of 32 and 64 µg/mL, respectively. In contrast, chlorhexidine (CHX), doxycycline (Dox), and sodium hypochlorite (NaClO) were more effective, while the guar extract showed the weakest inhibition; additionally, antibiofilm assessments were followed by antibacterial outcomes. However, cytotoxicity studies exhibited the least toxicity for MgO NPs compared with other compounds. The dental adherence test also showed that MgO NPs can inhibit bacterial interactions with the dental surface without inhibiting bacterial growth at sub‐MIC concentrations. Meanwhile, other groups killed them rapidly before they could adhere to teeth. Here, biocompatibility and long‐term antibacterial effectiveness were advantages of biogenic MgO NPs over other compounds that have been shown to be toxic to the host over long‐term consumption. Therefore, guar extract‐mediated MgO NPs demonstrated that they can be a favorable alternative for biofilm control in dental health without toxicity to related tissues in the oral cavity.

## 1. Introduction

Bacterial biofilms are the leading cause of dental caries, originating from a wide range of bacteria inhabiting the oral cavity. Building biofilm on the tooth surface drives bacterial colonization and is the onset of tooth decay [1]. In addition, biofilm formation provokes unpleasant breath and interferes with toothbrushing [2]. Among the biofilm‐producing pathogens, *Enterococcus faecalis* stands out with its high tendency to adhere to tooth surfaces [3]. Currently, the main challenge is microbial resistance to traditional antimicrobial treatments, which is the leading cause of the majority of bacterial infections [4]. Therefore, the increasing antibiotic resistance and the side effects of antimicrobial agents have raised the necessity for alternative, eco‐friendly, and biocompatible options for dental hygiene. In this line, nanoscale formulations and nanodrugs have attracted the attention of health researchers as a promising field [5].

Nanoparticles (NPs) exhibit extraordinary physicochemical and biological activity due to their small size and high surface‐to‐volume ratio. As a large class of nanomaterials, metal NPs have a wide range of applications in various industries, including chemistry, physics, and medicine. A variety of techniques can be applied to produce metal NPs. Metal NPs can exhibit unique properties for various applications depending on the synthesis routes. In most industries, metal NPs produced by chemical and physical processes are preferred [6].

However, attention has focused on metal NPs with biological origins in medicine. Studies have demonstrated that NPs produced by chemical methods have higher efficacy. In contrast, biogenic NPs are more biocompatible under biological conditions, having fewer side effects. Besides, due to the effective role of metabolites and natural compounds in the synthesis of NPs, a synergistic effect may be created in the biological activity of NPs [7, 8]. Magnesium oxide (MgO) NPs have many bioactivities, including antimicrobial, anti‐inflammatory, anticancer, antioxidant, and antidiabetic. Recent studies have confirmed the role of MgO NPs made with biogenic reactants and precursors in inhibiting wound infections and benefiting wound healing [9, 10]. Since bacterial biofilms create an impermeable structure against a wide range of antimicrobial drugs, NPs can disrupt biofilms in various ways due to their unique structure. MgO NPs have strong antibiofilm effects on a wide range of pathogens. In a study, the role of MgO NPs on several strains of pathogens resistant to various antibiotics was evaluated. These NPs demonstrated that they can inhibit bacterial growth and biofilm formation by multiple mechanisms [11]. The antimicrobial efficacy of MgO NPs is attributed to their ability to generate reactive oxygen species (ROS), disrupt bacterial cell membranes, and inhibit the formation of biofilms. These properties make them particularly suitable for dental applications, where preventing bacterial adherence and biofilm formation is critical for maintaining oral health. However, the effectiveness of MgO NPs in inhibiting dental pathogens, their biocompatibility, and their long‐term stability remain areas of active investigation [12]. Additionally, MgO NPs have garnered significant attention recently owing to their unique physicochemical properties, such as high stability, biocompatibility, and wide‐spectrum antimicrobial activity [13]. In this respect, the potential of medicinal plants for synthesizing metal NPs has been highlighted in drug design, offering different therapeutic effects [14]. Guar gum (*Cyamopsis tetragonoloba*) is a polysaccharide with a high ability to synthesize NPs. These polysaccharides can play a regenerative role in the synthesis process of metal NPs. In addition, guar polysaccharides can also play an effective role in the stabilization process of NPes as stabilizers and encapsulating agents [15]. Research has shown that NPs coated with biological materials have lower host toxicity properties and possess greater bacterial binding and microbial killing power [16]. In this study, an attempt has been made to synthesize MgO NPs using an aqueous extract of guar gum via a biological method and to investigate the biological effects related to dental hygiene, especially the inhibition of *E. faecalis* bacteria as an indicator of decreasing dental biofilm. Additionally, other bioactivities and therapeutic effects, such as antioxidant activity and cytotoxicity, were investigated with a comparative analysis of biogenic MgO NPs with conventional antimicrobial agents, such as chlorhexidine (CHX), doxycycline (Dox), and sodium hypochlorite (NaClO), which are essential for evaluating their potential as viable alternatives in dental care.

## 2. Materials and Methods

### 2.1. Chemicals and Experimental Equipment

Chemical materials were purchased from Merck Co. (Germany). DPPH (2,2‐diphenyl‐1‐picrylhydrazyl) and MTT [3‐(4,5‐dimethylthiazol‐2‐yl)‐2,5‐diphenyltetrazolium bromide] were provided by Sigma Company (USA). RPMI‐1640‐HS and fetal bovine serum (FBS) were procured from Kiazist (Iran). *E. faecalis* was supplied from the bacterial collection of Baharafshan Company (Tehran, Iran). Fibroblast‐like cell line (HGF1, Code: C165) was purchased from the Pasteur Institute cell bank (Pasteur Institute, Iran). All other materials were laboratory extra‐pure grade as received.

### 2.2. Preparation of Guar Gum Extract

To create the ethanol guar gum extract, 2 g of guar powder was added to 100 mL of 70% ethanol in a sealed 250 mL Erlenmeyer flask. The mixture was stirred completely on a magnetic stirrer until entirely suspended. The mixture was heated at 65 °C for 2 h in a water bath. After that, the solution was filtered using Whatman filter paper No. 1, and the collected filtrate was kept in the refrigerator for NP synthesis.

### 2.3. Biosynthesis of Magnesium NPs

Biogenic MgO NPs were synthesized using guar metabolites as reducing and stabilizing agents in ethanol extract. According to a protocol described by Suresh et al. [16], 2.56 g of Mg(NO_3_)_2_·6H_2_O (0.1 M) was dissolved in 60 mL of deionized water in a 250‐mL flask and placed on a magnetic stirrer, shaken at 150 rpm. After ensuring complete dissolution, 20 mL of the ethanol extract of guar gum was slowly added while the mixture was continuously stirred. The reaction mixture was maintained at 60 °C for 3 h until a yellowish‐white color appeared. After that, the solution was filtered and collected for centrifugation at 10,000 rpm for 15 min. Finally, the precipitate was dried in an oven at 80 °C for 12 h and then calcined in a furnace at 500 °C for 5 h [16].

### 2.4. Analytical and Physiochemical Studies of Mg NPs

Physicochemical features of biogenic MgO NPs were examined using instrumental techniques, including UV–visible spectroscopy for surface plasmon resonance (SPR) analysis, SEM imaging for morphological and size distribution studies, X‐ray diffraction (XRD) spectroscopy for crystallographic analysis, and FTIR spectroscopy for estimating possible functional groups involved in reducing and coating/stabilizing MgO NPs.

### 2.5. Antibacterial Studies

#### 2.5.1. MIC and MBC Determination

Antibacterial assessments were conducted on *E. faecalis* as one of the pathogenic indicators of dental caries. MIC values were determined with the microdilution method in a 96‐well plate. For this, 10 µL of fresh bacterial cells with a 0.5 McFarland cell density were inoculated in each well containing 100 µL of Muller‐Hinton broth (MHA) medium. Treatments included serial dilutions of 512–1 µg/mL for MgO NPs, guar extract, Dox, CHX, and NaClO. Equal volumes of each dilution (40 µL) were added to the wells and incubated at 37 °C for 24 h. MICs were determined based on the lowest concentration at which no visible bacterial growth was observed in the wells. To determine the MBC, bacterial samples were taken from MIC wells using a metal loop and streaked onto MHA plates. After 24 h of incubation, the lowest concentrations with no colonies were considered the MBC values for each treatment.

#### 2.5.2. Well Diffusion (WD) Assay

Antibacterial activities of MgO NPs and other compounds were examined using a WD assay method. For this, sterile MHA plates were prepared, and the bacterial cells were spread evenly across the surfaces of the agar plates in a lawn‐streak pattern. After that, a 6‐mm steel punch created five wells, and the wells were filled with 40 µL of test materials at the MIC concentration for each, as predetermined in the previous step. Finally, the plates were placed in a bacterial incubator at 37 °C for 24 h, and growth inhibition zones were determined using a digital ruler.

#### 2.5.3. Time Killing Assay

The antibacterial activities of MgO NPs and related groups were examined with regular exposure to an MHB medium. In this step, known bacterial cell densities (10^6^ CFU/mL) were treated with test compounds at their respective MIC values and incubated at 37 °C. Bacterial killing potentials were assessed by a serial dilution colony counting assay on MHA agar plated at regular time intervals (0, 0.5, 2, 5, 8, and 24 h).

### 2.6. Antibiofilm Studies

Antibiofilm activity of MgO NPs and other compounds was determined by growing *E. faecalis* in a 24‐well plate. For this, 0.5 mL of bacterial cells (1.5 × 10^8^ CFU/mL) was seeded in wells containing 1.5 mL of TSB medium supplemented with 1% glucose to induce biofilm formation. Treatments were performed by adding 1 mL of MgO NPs and other test materials to each well. After 48 h, the wells were emptied and washed gently with PBS buffer. The adhered bacterial biofilms were stained using 0.1% crystal violet (CV). The residual stain was discarded by washing, and then the biofilms were destained using 1% glacial acetic acid. Finally, CV absorbance was measured at 570 nm by a UV–visible spectrometer system (Genway, England). Finally, biofilm inhibition was calculated as follows:

Antibiofilm efficacy %=Control OD−Treated ODControl OD×100.

### 2.7. Dental Adherence Assay

The effect of MgO NPs and other treated groups on bacterial adherence to the dental surface was determined using a direct exposure method. In this experiment, the teeth were first washed with a normal saline solution and then treated with NaClO (25.5%). Further, to remove residual smear layers, the teeth were treated with EDTA (15%) and washed twice with ethanol and sterile distilled water. After that, 5 mL of TSB medium containing MIC concentrations of test compounds was added to glass tubes. One tooth was immersed in the medium, and a bacterial suspension (1.5 × 10^7^ CFU/mL) was inoculated into the glass tubes. After 48 h of incubation, the teeth were taken from the tubes and washed with PBS to remove non‐adherent bacteria. Finally, the biofilm formed on the dental surfaces was stained with 0.1% safranin.

### 2.8. Antioxidant Assay

The antioxidant activity of MgO NPs and gum extract was examined using a DPPH inhibition assay. First, a DPPH solution (0.1 mM in methanol) was prepared, and test compounds (50 µL) were treated with an equal volume of DPPH reagent (50 µL) in a 96‐well plate. The reaction mixture was incubated in the dark for 30 min, and absorbance was measured at 517 nm. Ascorbic acid was used as a positive control, achieving 100% DPPH inhibition. Finally, DPPH inhibition was calculated as an inhibition percentage using the following formula:

DPPH inhibition (%)=Absorbance of control (DPPH)−Absorbance of sample (antioxidant + DPPH)Absorbance of control (DPPH)×100.

### 2.9. Cytotoxicity Assay

The toxicity of MgO NPs, guar gum, Dox, and CHX was studied on normal HGF fibroblasts using the MTT assay. In this experiment, the cells (10^4^ cells/well) were seeded in the wells of a 96‐well plate containing 100 µL RPMI culture media supplemented with 10% FBS. Treatments were conducted by adding 50 µL of test compounds as serial dilutions of 0–100 µg/mL. The plate was incubated in 5% CO_2_ and 95% humidity for 24 h. After that, the wells were removed from the media, and adhered cells were treated with 100 µL of fresh media containing MTT reagent (10 µL). After 5 h of incubation, DMSO was added to the wells to dissolve the formazan precipitate, and absorbance was determined at 570 nm using an ELISA reader system. All treatments were performed in triplicate, and IC_50_ values were calculated based on mean ± SD using GraphPad Prism software version 8.`

## 3. Results and Discussion

### 3.1. Optical and Visual Confirmation of Biogenic MgO NPs

Biogenic MgO NPs were synthesized using ethanolic guar extract, which functioned as a reducing and stabilizing agent. Upon reaction of the guar metabolites with magnesium nitrate, the reaction mixture underwent a color transition from pale green to milky pale, a phenomenon attributed to light scattering effects caused by the nucleation and growth of NPs within the colloidal system (Figure 1a) [17]. The 12‐h duration promotes a controlled reduction mechanism, likely stemming from the moderate reduction activity of phytochemical constituents in the guar gum, such as hydroxyl and carboxyl groups within its polysaccharide matrix, which progressively reduced Mg^2+^ ions [14, 15]. As reported in prior studies, the observed color shift may correspond to the formation of magnesium hydroxide (Mg(OH)_2_) intermediates or transient precursors that undergo thermal decomposition during calcination to yield crystalline MgO NPs [18]. UV–visible spectroscopic analysis (200–600 nm range) revealed a prominent absorption band at 271 nm (Figure 1b), characteristic of the formation of MgO NPs. This spectral feature aligns with SPR profiles documented in analogous green synthesis studies utilizing biological‐derived agents, including *Pisonia alba* leaf extract (272 nm) [19], pumpkin seed extract (265.5 nm) [20], *Saccharomyces cerevisiae* extract (270 nm) [21], Neem Leave extract (273.5 nm) [22], thereby corroborating the successful biosynthesis of MgO NPs via guar gum‐mediated reduction.

Figure 1(a) Color change appearance of solution reaction during MgO NPs using guar extract and (b) UV–visible absorbance spectra of MgO NPs and guar extract.

**Figure:**
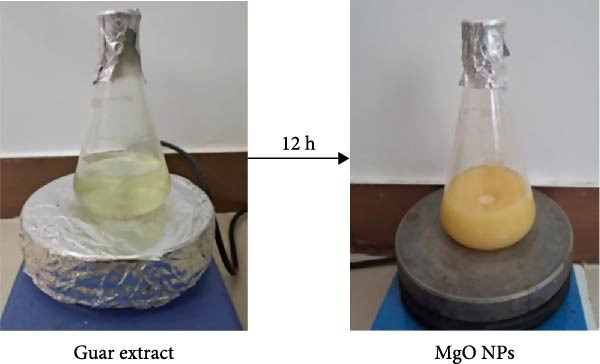
(a)

**Figure:**
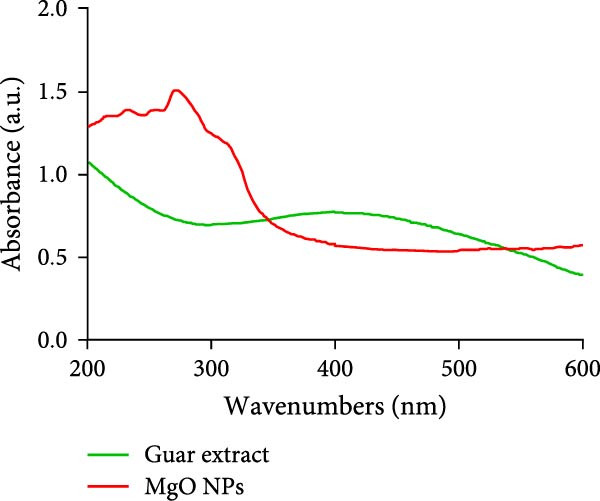
(b)

### 3.2. Morphological Studies by SEM‐Energy‐Dispersive X‐Ray Spectroscopy (EDS) and TEM Imaging

SEM imaging elucidated the morphological, compositional, and size dispersity of MgO NPs. Figure 2a shows the SEM image of MgO NPs synthesized using guar gum extract. The MgO NPs exhibited a flaky structure with partial aggregation, a typical morphology in biogenic nanomaterials attributed to their high surface energy, which caused them to cluster and reduce their surface area. Additionally, the TEM image of MgO NPs (Figure 2b) showed higher resolution, precise size confirmation, and clearer particle boundaries. The particle size distribution corresponding to the SEM image featured particle diameters ranging from 15 to 45 nm, with an average size of ~30 nm (Figure 2c). The size range below 100 nm is particularly significant for biomedical applications. Smaller dimensions enhance cellular uptake and increase the surface‐to‐volume ratio, resulting in higher reactivity and biological activity. Our findings are consistent with previous studies showing superior antimicrobial and photocatalytic performance of MgO NPs in this size range. In this regard, Ahmed et al. reported that biogenic MgO NPs with a size of 30 nm exhibited strong antibacterial activity against *Staphylococcus aureus* and *Escherichia coli*, as well as 90% photocatalytic degradation efficiency for methylene blue [23]. The size, shape, and composition of these NPs make them strong candidates for use in biomedical applications [24]. Another study confirmed that biogenic MgO NPs synthesized using *Taxus wallichiana* leaf extract, characterized by a spherical shape of about 20–30 nm, exhibited significant photocatalytic and biological activities [25]. Figure 2c,d demonstrates EDS mapping and elemental analysis, corroborating the high compositional purity of the synthesized MgO NPs with no impurities. This further validates the effectiveness of the guar gum‐mediated green synthesis approach. Additionally, the presence of signal peaks for carbon, nitrogen, and sulfur established a crucial involvement of bioactive metabolites from guar extract as reducing and stabilizing agents for Mg NPs formation [26].

Figure 2(a) SEM image of MgO NPs, (b) TEM image of MgO NPs, (c) size distribution of NPs based on the SEM image, (d) EDS of MgO NPs, and (e) mapping pattern of MgO NPs synthesized using guar extract.

**Figure:**
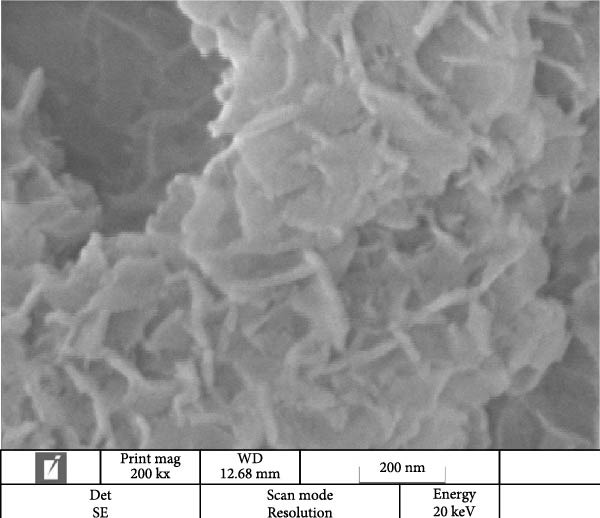
(a)

**Figure:**
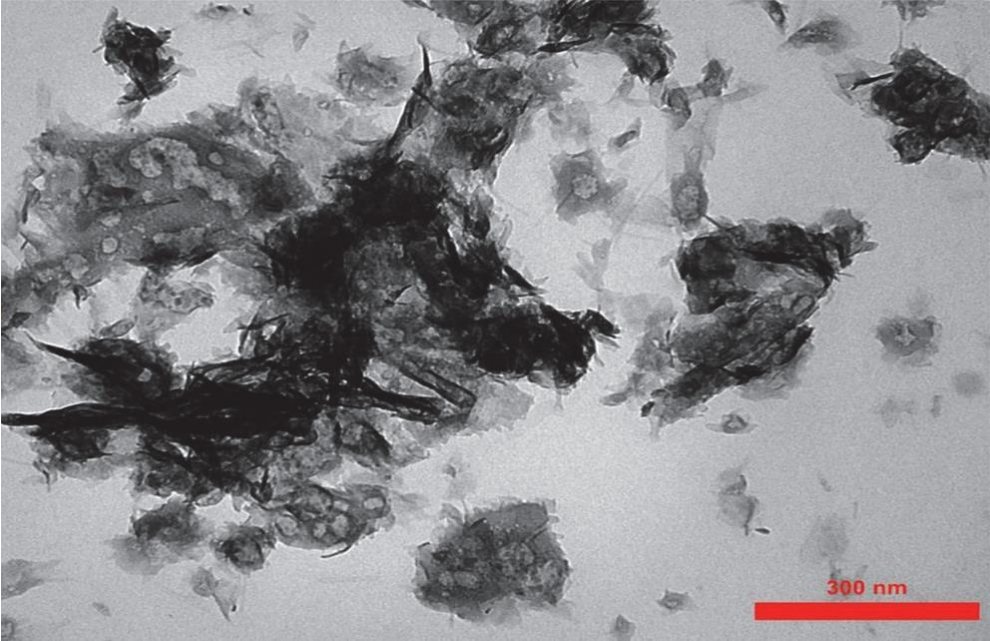
(b)

**Figure:**
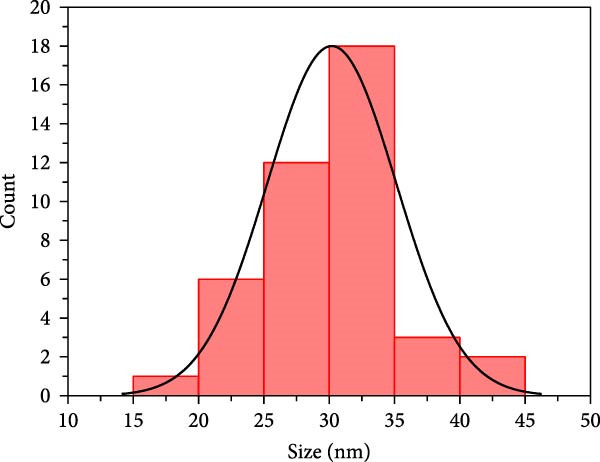
(c)

**Figure:**
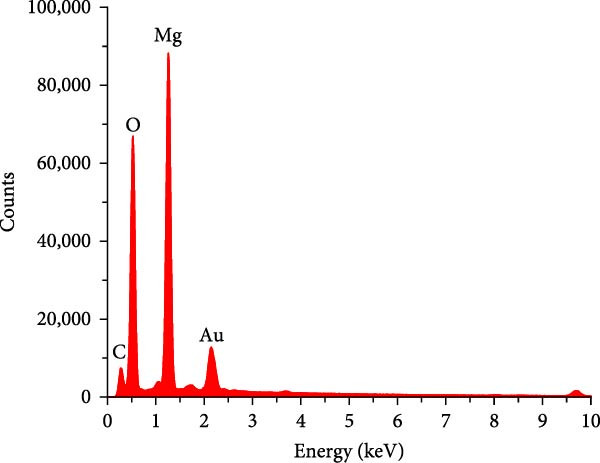
(d)

**Figure:**
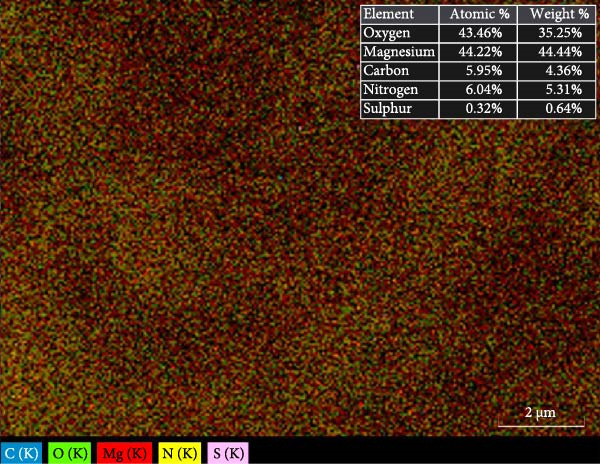
(e)

### 3.3. Crystallographic (XRD) Analysis of MgO NPs

**Figure 3:**
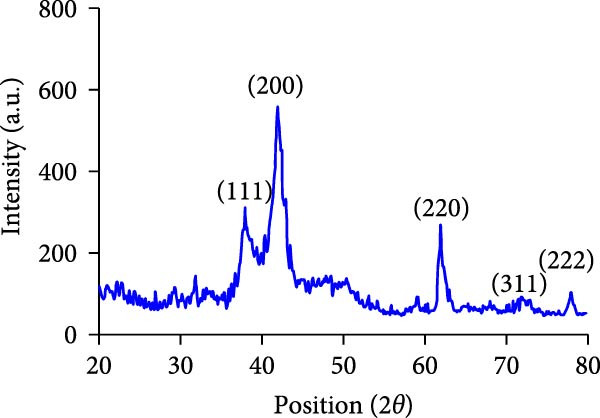
X‐ray diffraction (XRD) spectrum of MgO NPs precipitated by guar extract.

The crystallographic pattern of biogenic MgO NPs was evaluated using XRD analysis. As seen in Figure 3, the XRD spectra provide major intense peaks at 2*θ* values of 37.87°, 41.95°, 62.04°, 72.17°, and 78.04° with characteristic planes indexing to (111), (200), (220), (311), and (222), which corres pond to the cubic (FCC) periclase structure matched with the JCPDS card No. 89‐7746. The presence of sharp peaks implies a more homogeneous pattern, showing up to 85% crystallinity, which aligns with the expectations in controlled biosynthesis of MgO NPs. According to the Scherrer equation, the crystallite size of MgO NPs was estimated to be 16.5 nm. The synthesized magnesium NPs demonstrate a relatively optimistic purity, opening up exciting possibilities for their investigations in biological activities.

### 3.4. FTIR Analysis

**Figure 4:**
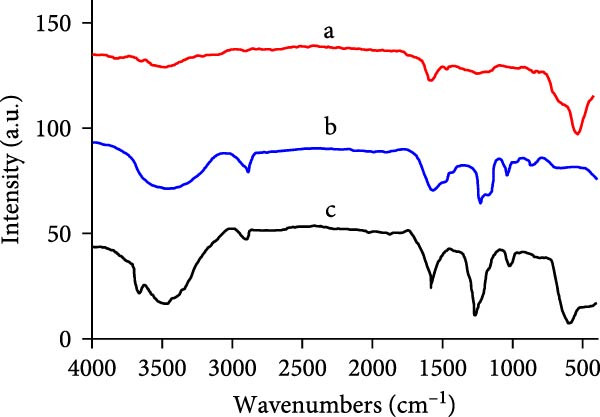
The FTIR spectra of (a) calcined MgO NPs, (b) guar extract, and (c) uncalcined MgO NPs.

The FTIR spectra of ethanolic guar extract and the corresponding MgO NPs provided insights into the functional groups involved in NP formation, coating, and stabilization. In Figure 4, the broad peak observed in the 3456.9 cm^−1^ region corresponds to O─H or N─H stretching vibrations, attributed to hydroxyl or amine groups in guar extract, which are crucial in reducing and stabilizing MgO NPs [27]. The peaks in the 2902.8 cm^−1^ range correspond to the C─H stretching of aliphatic compounds, with reduced intensity after NP synthesis in the MgO NPs spectrum (2908.3 cm^−1^) [28]. A peak at 1575.2 cm^−1^, attributed to C═O stretching of carbonyl groups and C═C vibrations from aromatic compounds, shifts to 1586.2 cm^−1^ in the MgO NP spectrum, indicating the role of polyphenols in the Mg ion reduction. The stretching peak in the 1246.0 cm^−1^ region is also associated with C─O functional groups involved in MgO NPs formation. A stretching peak around the 1043.1 cm^−1^ region can correspond to the C─O─C vibration mode in glycosidic bonds, shifting to 1021.1 cm^−1^ in the MgO spectrum. Hence, this presence highlights the role of polysaccharides in stabilizing NPs by adsorption on the surface of MgO NPs. In spectrum‐b, diminished peaks and several chemical shifts were observed in the uncalcined MgO NPs. Moreover, a unique peak in the 593.2 cm^−1^ region is also related to the Mg─O vibrational bond, validating the effective creation of MgO NPs [29]. In contrast, in spectrum‐c, the organic functional groups present in the guar extract, such as O─H, C─H, and C═O, were notably reduced or disappeared due to the thermal decomposition of the organic capping agents following calcination. Additionally, a prominent Mg─O absorption peak appeared at 512 cm^−1^, signifying the yield of pure, crystalline MgO NPs.

### 3.5. Antimicrobial Assay

The antibacterial efficacy of MgO NPs synthesized using guar extract was examined against *E. faecalis* and compared with guar extract alone, Dox, CHX, and NaClO as standard controls. Figure 5 shows a qualitative view of antimicrobial experiments. In addition, the calculated results presented in Table 1 provide a quantitative assessment of the antibacterial properties of the test materials, including MIC, MBC, time‐kill (TK) assay, and zone of growth inhibition (ZGI) measurements. The MIC values for MgO NPs and NaClO were determined to be 32 µg/mL. In contrast, Dox and CHX exhibited lower MIC values of 16 µg/mL. However, the MBC values revealed that MgO NPs required a higher concentration (64 µg/mL) to achieve bactericidal effects compared to Dox, CHX, and NaClO, all of which had MBC values of 32 µg/mL. These results demonstrate that MgO NPs effectively inhibit bacterial growth at higher concentrations, thereby eradicating *E. faecalis*. In this regard, Rodríguez et al. [13] reported that MgO NPs exhibited the most outstanding inhibition at a concentration of 20 mg/mL against biofilm‐resistant strains of *S. aureus* and *S. mutans*. In the study by Hayat et al. [11], the antibacterial activity of MgO NPs was found to cause cytoplasmic leakage in bacterial pathogens. They reported greater cellular protein leakage in Gram‐negative bacteria. Studies have shown that MgO NPs have another advantage besides their desirable antimicrobial effects: their high biocompatibility [30]. Although multiple studies have been conducted on the antimicrobial activity of biogenic MgO NPs, their effective role on oral and dental pathogens, especially *E. faecalis*, has been limited [31]. Time exposure can be critical for controlling oral infections, so time‐killing kinetics were evaluated for the test materials. In this step, the effect of time on the killing efficacies of CHX was to have the fastest bactericidal action, with 99% killing in less than 5 min, followed by NaClO (30 min). In contrast, this value was determined for MgO NPs and Dox, which have nearly 2 h of exposure time, resulting in the killing of up to 90% of bacterial colonies. These findings align with previous studies that have reported CHX and NaClO to be potent antibacterial agents with rapid action, particularly in endodontic applications [32, 33]. However, the relatively slower action of MgO NPs may be attributed to their mechanism of action, which involves the generation of ROS and the gradual disruption of bacterial cell membranes [34, 35].

Figure 5(a) MIC values for MgO NPs, guar gum extract, doxycycline (Dox), chlorhexidine (CHX), sodium hypochlorite, and a negative control without drug were obtained separately at two‐folds different dilutions. (b) The growth inhibition zone based on the well diffusion (WD) agar method obtained for all groups, and (c) MBC obtained based on MIC values of different groups for *E. faecalis bacterium*.

**Figure:**
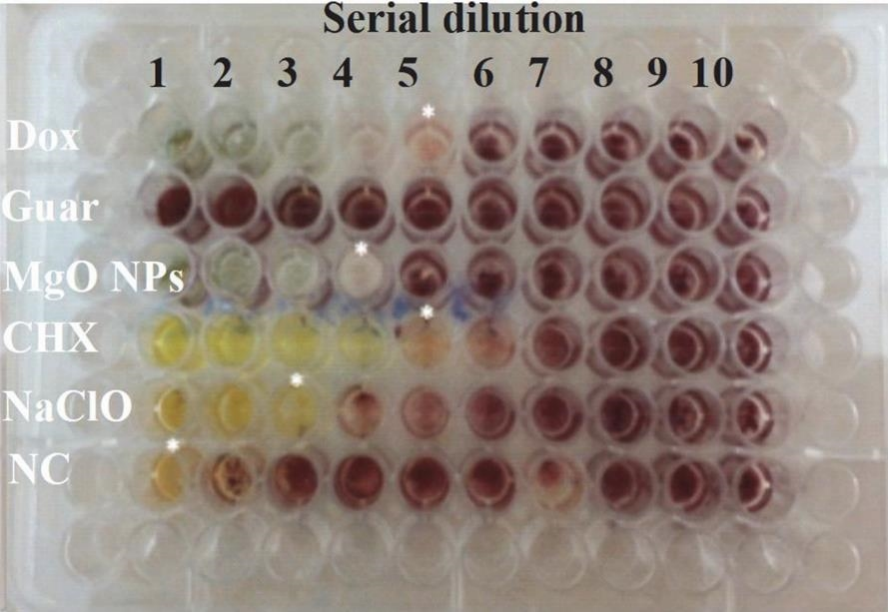
(a)

**Figure:**
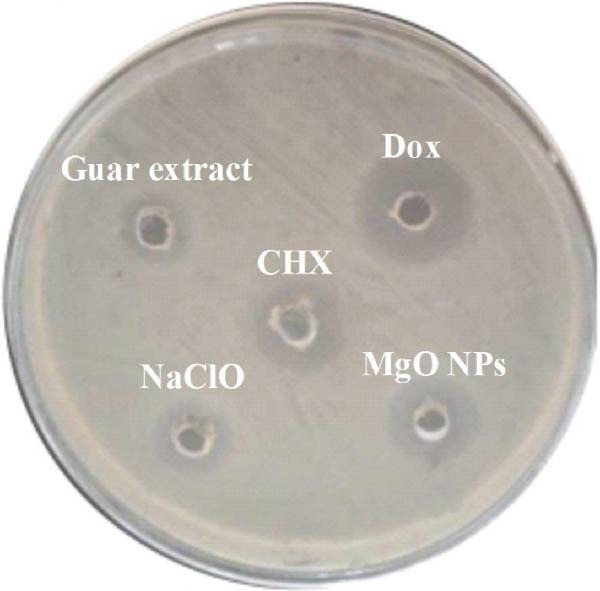
(b)

**Figure:**
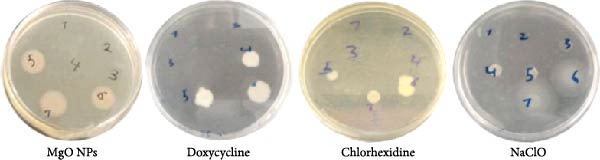
(c)

**Table 1:** Antibacterial results obtained by different methods.

Treatments	Antibacterial outcomes			
	MIC (µg/mL)	MBC (µg/mL)	TK (h)	WDA (mm)
MgO NPs	32	64	2	16.86 ± 2.31
Guar extract	—	—	—	8.31 ± 1.04
Dox	16	32	2	18.41 ± 2.83
CHX	16	32	<5 min	12.95 ± 2.51
NaClO	32	32	30 min	12.07 ± 2.11

In terms of the WD test, MgO NPs showed a significant antibacterial effect with an inhibition zone of 2.31 mm, comparable to Dox (2.83 mm) and superior to CHX (12.95 mm) and NaClO (12.95 mm). Literature has proven that several factors may affect the diffusion of antimicrobial agents in agar plates. Therefore, methods based on the release of agents in agar structure to evaluate the antimicrobial effects of NPs alone may not reveal their antimicrobial potency [36, 37]. Considering the findings of the time‐killing test and MIC and WD methods, a series of contradictions was observed. These discrepancies can be attributed to the trial conditions and the duration of contact with antimicrobial materials on the bacterial cells. Literature has demonstrated that the antimicrobial efficacy of materials, especially NPs, is significantly correlated to their size, solubility, and surface charge. There may be differences in the results of rapid tests compared to long‐term tests, such as diffusion methods [38].

### 3.6. DPPH Scavenging Assessment

The antioxidant activity of MgO NPs was thoroughly examined, and the results were compared with those of ascorbic acid and guar extract. The results were presented as a DPPH scavenging percentage for all compounds. According to Figure 6a, MgO NPs, gum extract, and ascorbic acid showed DPPH inhibitory concentration with IC50 values of 14.08, 11.5, and 8.27 mg/ml, respectively. Therefore, calculations showed that guar extract had more antioxidant activity than MgO NPs at all concentrations, while ascorbic acid demonstrated the highest inhibition power. Considering the antioxidant capacity of MgO NPs, it can be implied that metabolite coating improves biocompatibility and modulates the side effects of most metal NPs. In addition, most infections can cause inflammation by stimulating the production of free radicals by the immune system and host cells. Therefore, MgO NPs might reduce inflammation and subsequent bacterial infections. In this regard, studies have established anti‐inflammatory potential for green‐synthesized MgO NPs. In this regard, Shahid et al. [39] synthesized MgO NPs using Alstonia scholaris extract, which showed a potent anti‐inflammatory effect in vitro compared with diclofenac sodium. Their findings revealed that biosynthesized MgO NPs inhibited 76.59% of albumin denaturation, indicating an inflammatory response in vitro [39].

Figure 6(a) DPPH scavenging assay for guar extract, MgO NPs in comparative mode with ascorbic acid. (b) Cell viability study on HF cells exposed to test materials.

**Figure:**
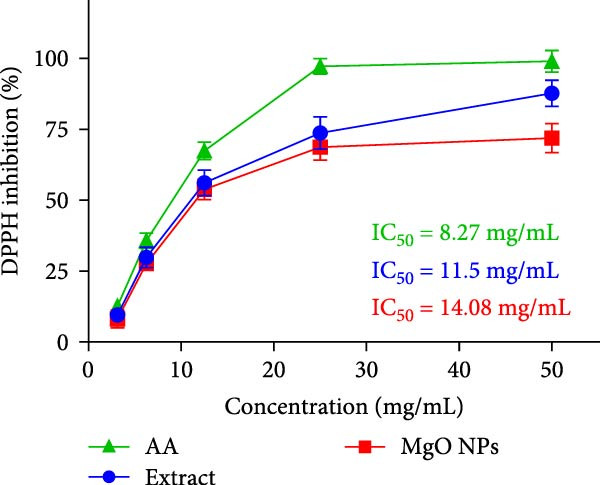
(a)

**Figure:**
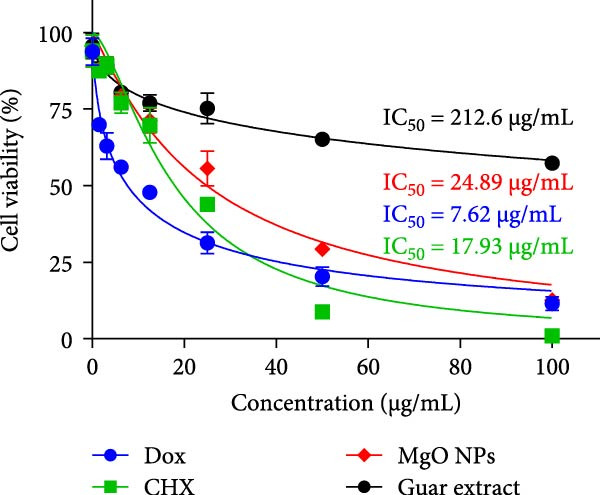
(b)

### 3.7. Cytotoxicity Assessment

The cytotoxicity of MgO NPs was examined on normal HF fibroblast cells using the MTT assay. Figure 6b demonstrates cell viability status at various MgO NPs concentrations (0–100 µg/mL). As anticipated from other studies, MgO NPs exhibited a dose‐dependent effect on cell survival. For comparisons between test materials, the IC_50_ values were determined to be 24.89, 212.6, 17.93, and 7.62 for MgO NPs, guar extract, CHX, and Dox, respectively. Based on the results, the cytotoxic effect of MgO NPs was moderate compared with CHX and Dox. Moreover, some studies suggest that MgO NPs have a protective effect on tissues affected by toxicity. In this line, Venkatappa et al. [40] showed that MgO NPs synthesized from *Tarenna asiatica* fruit showed promising protection against oxidative stress‐induced tissue damage and thrombosis. Regarding the compatibility of MgO NPs, Verma et al. [41] demonstrated that MgO NPs synthesized using *Calotropis gigantea* extract showed higher biocompatibility in embryonic zebrafish (*Danio rerio*). Therefore, MgO NPs not only exhibited the desired biocompatibility but also demonstrated a therapeutic effect on damaged tissues. Our study showed satisfactory survival for normal cells treated with MgO NPs synthesized using guar extract.

### 3.8. Antibiofilm Activity Assessment

Figure 7 illustrates a 24‐well plate showcasing the biofilm formation process in response to the tested materials, along with the MIC values derived from previous experiments. After staining with 0.1% CV, the reduction of biofilm formation was evaluated by measuring the absorbed CV using the spectrophotometric method described above. According to Figure 6b, the percentage of biofilm inhibition in the high‐dose treatment (200 µg/mL) of MgO NPs, Dox, and CHX exhibited 90% inhibition without significant differences. However, this inhibition varied when bacterial biofilms were treated with 50 µg/ml of the three materials. Although the biofilm inhibition mechanisms of these materials differed, the outcomes were promising, with potential for eradicating bacterial biofilms. In various studies, MgO NPs have been shown to disrupt microbial biofilms through multiple mechanisms. Hayat et al. [11] demonstrated that MgO NPs were potent antibiofilm agents against *S. aureus* and *E. coli* at concentrations of 250 and 125 µg/mL, respectively, with inhibition efficacies of 82.1% and 82.1%. They concluded that antibiofilm activity could be attributed to the strong affinity of Mg ions to the biofilm matrix, disrupting its integrity. Therefore, depending on the type of pathogen strain, MgO NPs may influence biofilm formation differently. Although studies have highlighted the antibiofilm activity of MgO NPs, the role of green‐derived capping or coating/stabilizing agents can be more critical to their bioactivities [31, 42]. In this regard, Younis et al. [43] demonstrated that MgO NPs synthesized by *Rosa floribunda charisma* extract exhibited antibiofilm efficiencies against three skin pathogens, such as *S. epidermidis*, *S. pyogenes*, and *P. aeruginosa*. Additionally, Mubarak Ali et al. [44] investigated the antibiofilm potential of chemically synthesized MgO NPs with varying potencies. They stated that MgO NPs exhibited the most prominent inhibition against *K. pneumoniae*, at ~80.89%, and the weakest against *E. coli*, at ~30%. In contrast, its inhibitory effect was moderate on Gram‐positive bacteria, such as *Staphylococcus* sp., *Micrococcus* sp., and *S. agalactiae*, with an effect of about 50%–70%. Here, the critical role of bioactive compounds involved in NPs formation is increasingly highlighted. Hence, the study by Fatiqin et al. [45] revealed that MgO NPs synthesized using *Moringa oleifera* leaf extract exhibited greater inhibition against Gram‐positive bacteria compared to Gram‐negative bacteria. In our study, the antibiofilm inhibition of MgO NPs synthesized using guar gum extract was satisfactory and as anticipated.

Figure 7(a) Antibiofilm assay of test materials against *E. faecalis* on 24‐well plate stained by crystal violet (0.1%). (b) Quantitative biofilm inhibition in exposure to different test materials concentrations (0–200 µg/mL).

**Figure:**
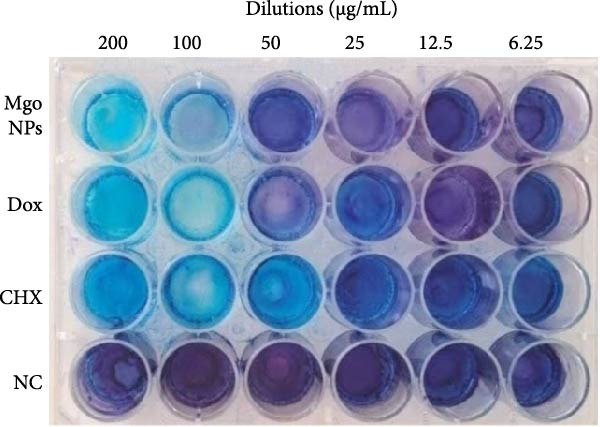
(a)

**Figure:**
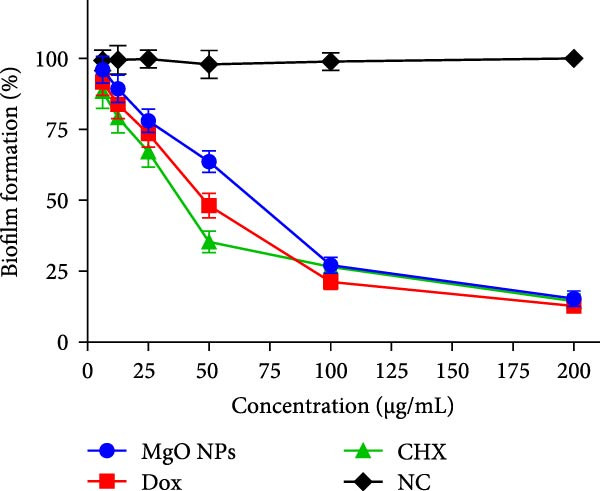
(b)

### 3.9. Bacterial‐Dental Adherence Assay

**Figure 8:**
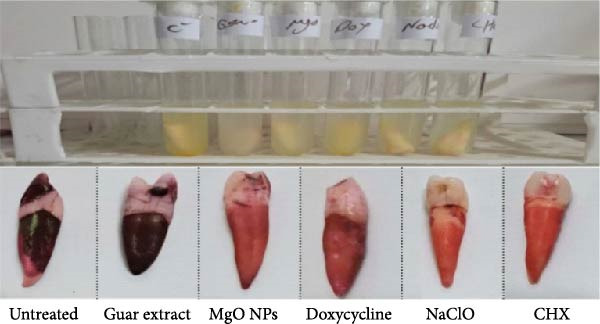
Antiadhesive assay of test materials against *E. faecalis* on dental surface stained by safranin (0.1%).

The antiadherence potential of MgO NPs, guar extract, and antibacterial controls was examined on dental surfaces after exposure to a single dose of each one. Figure 8 illustrates glass tubes containing bacterial culture in contact with the tooth. The treated teeth were stained with 0.1% safranin to detect adherent bacteria, as described in Section 2. Among the test groups, it was elucidated that those treated with CHX, Dox, and NaClO had the highest bacterial density on dental surfaces. After that, the MgO NPs‐treated groups showed an antiadherent potential greater than that of guar extract. The results revealed that the adherence inhibitory effect of MgO NPs was moderate compared to antiseptics, CHX, NaClO, and the antibiotic Dox.

Additionally, studies have demonstrated that MgO NPs can inhibit adhesion factors in pathogens and reduce colonization on the dental surface, even though they are unable to kill planktonic bacteria. Wetteland et al. [46] examined the effect of MgO NPs on the ability of bone marrow‐derived mesenchymal stem cells infected with *E. coli* and *S. epidermidis*. They found that MgO NPs at a 200 μg/mL dose offer dual functions of promoting BMSC proliferation and reducing bacterial adhesion on the scaffolds. Therefore, they suggested that MgO NPs have the potential for use in the production of medical implants. In the present study, other compounds demonstrated higher antiadhesion activity than MgO NPs. However, considering their high host toxicity, MgO NPs may still be a preferable option due to their promising inhibitory effects on dental adhesion, desirable biocompatibility, and moderate cytotoxicity.

## 4. Conclusion

The experiments demonstrated that MgO NPs synthesized using guar extract are promising as an antibacterial agent, particularly when long‐term inhibition of bacterial colonization is required. Although they may not respond as quickly as some conventional antibacterial agents, their broad efficacy, biocompatibility, and lower host toxicity may make them suitable for various biomedical and environmental applications. This study confirmed that MgO NPs effectively reduced the colonization of *E. faecalis* on the tooth surface. Taken together, these biogenic MgO NPs could be developed into oral and dental disinfectant products. However, comprehensive trials are crucial to improve their long‐term stability, biocompatibility, and potential combination with other antibacterial agents.

## Disclosure

All the authors gave their consent to publish this research project.

## Conflicts of Interest

The authors declare no conflicts of interest.

## Author Contributions

**Asma Sepahdar**: investigation, writing – original draft. **Matin Kordestani, Saeed Bahadori, and Maryam Karkhane**: investigation, formal analysis. **Suresh Ghotekar and Pegah Shakib**: conceptualization, data analysis. **Abdolrazagh Marzban**: conceptualization, supervision, writing – review and editing.

## Funding

The study received financial support from Lorestan University of Medical Sciences with Grant Number 23/7/1403 and ethical approval code: IR.LUMS.REC.1403.308.

## Data Availability

The datasets that support the findings of this study are available upon request from the corresponding author. The data are not publicly available due to privacy or ethical restrictions.
